# Validation of the global lung initiative 2012 multi-ethnic spirometric reference equations in healthy urban Zimbabwean 7–13 year-old school children: a cross-sectional observational study

**DOI:** 10.1186/s12890-020-1091-4

**Published:** 2020-02-28

**Authors:** Tafadzwa Madanhire, Rashida A. Ferrand, Engi F. Attia, Elopy N. Sibanda, Simba Rusakaniko, Andrea M. Rehman

**Affiliations:** 1 University of Zimbabwe, Harare, Zimbabwe; 2 Biomedical Research and Training Institute, 59 Pendennis Road, Mt Pleasant, Harare, Zimbabwe; 3 Department of Clinical Research, London School of Hygiene and Tropical Medicine, London, UK; 4 Department of Medicine, Division of Pulmonary, Critical Care and Sleep Medicine, University of Washington, Seattle, WA USA; 5 National University of Science and Technology, Bulawayo, Zimbabwe; 6 MRC Tropical Epidemiology Group, London School of Hygiene and Tropical Medicine, London, UK

**Keywords:** Pulmonary function, Africa, Spirometry

## Abstract

**Background:**

The 2012 Global Lung Function Initiative (GLI_2012_) provide multi-ethnic spirometric reference equations (SRE) for the 3–95 year-old age range, but Sub-Saharan African populations are not represented. This study aimed to evaluate the fit of the African-American GLI_2012_ SRE to a population of healthy urban and peri-urban Zimbabwean school-going children (7–13 years).

**Methods:**

Spirometry and anthropometry were performed on black-Zimbabwean children recruited from three primary schools in urban and peri-urban Harare, with informed consent and assent. Individuals with a history or current symptoms of respiratory disease or with a body mass index-z score (BMI) < − 2 were excluded. Spirometry z-scores were generated from African-American GLI_2012_ SRE, which adjust for age, sex, ethnicity and height, after considering all GLI_2012_ modules. Anthropometry z-scores were generated using the British (1990) reference equations which adjust for age and sex. The African-American GLI_2012_ z-score distribution for the four spirometry measurements (FVC, FEV_1_, FEV_1_/FVC and MMEF) were evaluated across age, height, BMI and school (as a proxy for socioeconomic status) to assess for bias. Comparisons between the African-American GLI_2012_ SRE and Polgar equations (currently adopted in Zimbabwe) on the percent-predicted derived values were also performed.

**Results:**

The validation dataset contained acceptable spirometry data from 712 children (344 girls, mean age: 10.5 years (SD 1.81)). The spirometry z-scores were reasonably normally distributed, with all means lower than zero but within the range of ±0.5, indicating a good fit to the African-American GLI_2012_ SRE. The African-American GLI_2012_ SRE produced z-scores closest to a normal distribution. Z-scores of girls deviated more than boys. Weak correlations (Pearson’s correlation coefficient < 0.2) were observed between spirometry and anthropometry z-scores, and scatterplots demonstrated no systematic bias associated with age, height, BMI or socioeconomic status. The African-American GLI_2012_ SRE provided a better fit for Zimbabwean paediatric spirometry data than Polgar equations.

**Conclusion:**

The use of African-American GLI_2012_ SRE in this population could help in the interpretation of pulmonary function tests.

## Background

Spirometry is a clinical tool used to measure and monitor lung function. There are well-defined spirometric variables that inform about patterns of lung function abnormalities and aid in the diagnosis of different types of lung disease that may manifest with obstructive and restricted lung function patterns [1]. Lung function results obtained from a patient after a spirometry manoeuvre are compared to appropriate spirometric reference equations (SRE) derived from healthy individuals of the same ethnicity, height, age, and sex [2]. SRE have traditionally been generated using different methods and populations, resulting in significant variability, and rarely including data from sub-Saharan Africa [3–6]. There is also increasing concern over the use of fixed percentage predicted cut-offs in SRE in clinical settings to define abnormalities as it can lead to incorrect interpretation of spirometry results [2, 7].

To address this, the European Respiratory Society (ERS), through the Global Lung Function Initiative (GLI), developed global SRE for healthy individuals aged 3–95 years in 2012. The data used to generate the GLI_2012_ SRE were collected from Europe, Australia, Latin America, East Asia, India, North America and North Africa [8]. The GLI_2012_ provide ethnic-specific equations for Caucasians, African-Americans, South East Asians and North East Asians. The GLI_2012_ provide age-, height-, sex- and ethnic-specific SRE [9]. These equations provide lower-limit-of-normal (LLN) values, which can be defined as the 5th percentile values (z-score < − 1.64) of the healthy, non-smoking population [2]. The z-score reflects the number of standard deviations a measurement is positioned from its predicted/reference value, centered at zero [10]. It is a function of a normally distributed population and is thought to be a more valid measure to define the LLN as compared to traditional fixed cut-offs (i.e., 0.8 for forced vital capacity [FVC] and forced expiratory volume in 1 s [FEV_1_], and 0.7 for the FEV_1_/FVC ratio) used to help define airflow limitation and obstruction [2, 11, 12]. Use of the GLI_2012_ SRE is endorsed by the American Thoracic Society (ATS) and the ERS, and many manufacturers now install the module in their devices [8, 13, 14].

Studies validating the GLI_2012_ SRE have made varying conclusions, with some indicating a poor fit for local populations [10, 15]. However, the FEV_1_/FVC ratio has consistently demonstrated a better fit across populations than other lung function measurements [10, 15–17]. Potential reasons for poor fit of SRE include sampling which is unrepresentative of the population, potential mis-specification of the prediction equations, and environmental factors such as exposure to indoor and/or ambient air pollution, malnutrition, and low socioeconomic status (SES), which may result in lower lung volumes on a population level, leading to erroneous estimations [18–23]. Like many SRE, the GLI_2012_ SRE lack contribution of lung function data from sub-Saharan African populations, and use of the African-American GLI_2012_ SRE is generally recommended for African populations [8].

As such, an ERS task force recommended additional studies to validate the GLI_2012_ SRE in non-Caucasian populations [8]. A cross-sectional observational study was performed to evaluate the performance of the GLI_2012_ SRE among urban and peri-urban Zimbabwean children aged 7–13 years. The GLI_2012_ SRE were also compared against the Polgar equations because they are currently used in clinical practice.

## Methods

### Study population

Between June and October 2018, black-Zimbabwean children aged 7 to 13 years were recruited from three schools in Harare randomly selected from three economic zones classified as high, medium and low-income status by the Ministry of Education. The schools were classified after taking into account the location and economic status of the school. Children were excluded from the validation dataset if they had a history of chronic respiratory disease or respiratory symptoms including cough with or without sputum, wheeze and shortness of breath in the past 3 months, or reported regular exposure to smoke in the past 6 months (living at least 3 days per week with people smoking cigarettes) [24, 25]. Children with body mass index (BMI) z-score < − 2 were also excluded from the analysis dataset [8, 26]. Eligible children were randomly selected from each class level in a 1:1 sex ratio in advance using class attendance registers supplied by the schools and replacements for those absent were conveniently sampled from the same class. Based on GLI guidelines, a minimum sample size of 150 was required for each group (boys and girls) to evaluate the GLI_2012_ SRE [27].

### Data collection

A self-administered parental paper questionnaire was used to collect data on children’s respiratory health, including asthma or other chronic respiratory diseases. An interviewer-administered paper questionnaire was used to record sociodemographic data and current respiratory symptoms from the children. Height (cm) and weight (kg) were measured barefoot in light clothing with 1.0 cm and 0.1 kg precision. A Seca mechanical medical weight scale and Seca 213 stadiometer (Seca Mechanical Floor Scales Class III, Seca Precision for health, Hamburg, Germany) were used to measure weight and height respectively. Spirometry was performed using Windows 10 *Koko S*_*x*_ software connected to a pneumotach (Koko Legend S_x_, nSpire Health, Inc. Longmont, USA*)* according to ATS/ERS guidelines [28].

The instructor demonstrated an exemplary spirometry manoeuvre before the child attempted spirometry. The test was phased as an initial deep breath, followed by a maximum exhalation phase and a final inhalation phase as per the instructor’s direction. Tests were performed from a standing position with each child taking on average 8–11 min to perform at least three volume-time curves. Children performed three to eight efforts and the best manoeuvre was used for analysis [28]. The best effort of manoeuvres was defined as the largest sum of FVC and FEV_1_ within 0.15 l (FVC > 1.0 l) and 0.1 l (FVC ≤ 1.0 l) of each other after considering the time of exhalation [29].

All volume-time curves were first checked by the diagnostic software, assessing the longevity of the exhalation phase (≥ 6 s in ≥10 year-olds and ≥ 3 s in < 10 year-olds) [30]. The operator further checked the degree of effort as indicated by the curve’s sharp peak, and absence of cough/glottic closure during exhalation. Only measures from children performing at least three acceptable and repeatable efforts were included in the validation dataset [28]. The same device was used for all spirometry sessions performed and the machine was calibrated daily before use and after a change in ambient conditions (two units change in temperature measured in degrees Celsius and atmospheric pressure measured in millimetres of mercury).

### Statistical analysis

Data was de-identified by unique identifier codes and entered into STATA for analysis (StataCorp. 2017. Stata Statistical Software: Release 15. College Station, TX: StataCorp LLC). Spirometry outcomes were FVC, FEV _1,_ FEV_1_/FVC ratio and MMEF (maximal-mid expiratory flow). GLI z-scores and LLN values for FVC, FEV_1_, FEV_1_/FVC, and MMEF, were computed using GLI_2012_ SRE using height, age, sex and ethnic data [2, 31]. The z-score and LLN values were calculated using the available Microsoft-Excel Macro calculators, which provide an age, height, sex and ethnic-specific value [8]. The GLI_2012_ z-score is an unbiased estimate showing the positioning of an observed spirometry value in the distribution of the GLI_2012_ SRE [32]. If the GLI_2012_ SRE and the observed spirometry values are in perfect agreement, the mean z-score is zero with a standard deviation (SD) of one (a normally distributed set of data). According to the consensus reached by the GLI team and other studies validating these SRE, a mean z-score outside the range of ±0.5 is considered to be clinically significant, corresponding to at least 5–6% difference in the specified lung function measurement [8, 10, 15–17]. The LLN was considered as the fifth percentile of the healthy population calculated using the GLI_2012_ SRE. We considered all GLI ethnic modules to determine if the African-American ones provided the most appropriate fit.

The Shapiro-Wilk test and visual plots (histograms and quantile-quantile (Q-Q) plots) were used to assess normality of variables. Outcomes were compared graphically against age, height, weight and BMI z-scores, calculated using the 1990 British reference values as well as school (as a proxy for SES) to determine if any bias was present [33]. A circular scatter around the origin would provide no evidence for bias with anthropometry z-scores, while no linear relationship should be present with age.

We also evaluated the association between anthropometry and spirometry z-scores using Pearson’s product-moment correlation and linear regression. A lack of correlation or association indicates a good fit of the GLI_2012_ SRE on the population [16].

The predicted GLI_2012_ were also statistically compared against the Polgar SRE for the observed measurements [34].

Normally-distributed variables are presented as mean (SD), and the student’s t-test was used to compare means of spirometry and anthropometry z-scores across demographic factors. All results are sex-specific to account for smaller lung volumes in girls compared to boys and the high variation expected in this age group of 7–13-year-olds, because girls will be at a more advanced stage of puberty than boys [35].

## Results

**Fig. 1:**
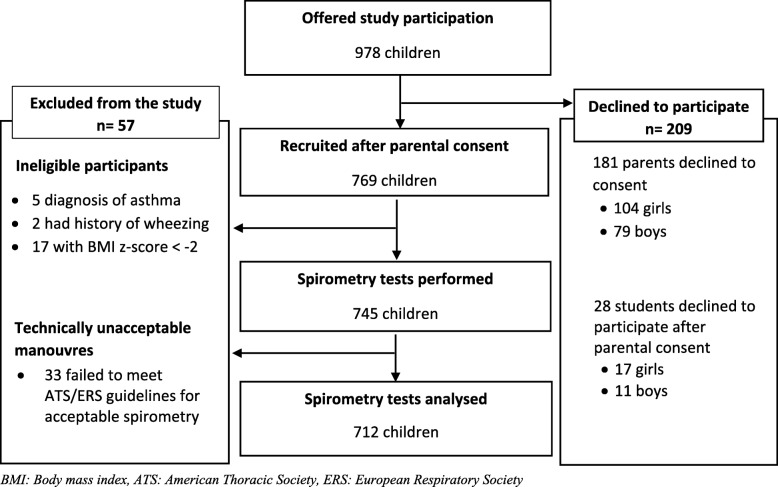
Participant recruitment flow-chart

Of 978 children that were approached, 209 (21%) did not provide consent. After exclusion of 24 individuals who did not meet eligibility criteria and 33 children who failed to perform technically acceptable spirometry measurements, 712 were included in the analysis (Fig. 1).

Age ranged from 7 to 13 for both girls and boys. However, boys had a higher mean age, BMI-for-age and MMEF z-scores, congruent to other studies [36–38] (Table 1).

**Table 1:** Characteristics of study participants by sex

Variables	Total (*n* = 712)	Boys (*n* = 368)	Girls (*n* = 344)	*p*-value
	mean (SD)	mean (SD)	mean (SD)	
Age (years)	10.5 (1.81)	10.7 (1.86)	10.3 (1.74)	0.005
Height (cm)	139.9(10.36)	140.2(10.25)	139.6(10.49)	0.410
Weight (kg)	34.4(7.73)	34.8(7.69)	34.0(7.67)	0.156
BMI (kg/m^2^)	17.4(2.09)	17.5(2.09)	17.2(2.09)	0.036
Height for age z-score	−0.15 (0.98)	− 0.24 (0.95)	− 0.05 (1.01)	0.005
Weight for age z-score	−0.02 (0.89)	0.01 (0.91)	−0.05 (0.87)	0.162
BMI for age z-score	0.07 (0.90)	0.19 (0.92)	−0.05 (0.86)	< 0.001
FVC z-score	−0.11 (1.01)	−0.08 (0.97)	− 0.15(1.05)	0.302
FEV_1_ z-score	− 0.36 (0.95)	−0.33(0.90)	− 0.40(0.99)	0.305
FEV_1_/FVC z-score	− 0.42 (1.16)	−0.42(1.11)	− 0.43(1.21)	0.916
MMEF z-score	− 0.56 (1.05)	−0.48(1.06)	− 0.66(1.02)	0.018

Spirometry z-scores (FVC, FEV_1_, FEV_1_/FVC, and MMEF) z-score were generated from African-American GLI_2012_; *p*-value from t-test showing the comparisons of z-score values between boys and girls participants; *p*-value = 0.123 from chi-square test of independence between sex and school income level (High (23.6), Middle (39.6), Low (36.8))

*FVC* Forced vital capacity, *FEV*_*1*_ Forced expiratory volume in one second, *FEV1/FVC* Ratio of FEV_1_ to FVC, *MMEF* Maximal mid-expiratory flow, *BMI* Body Mass Index, *SD* Standard Deviation

On average, children who were excluded from the study were older (11.6 years, SD: 1.45), than those considered for analysis. The ratios of boys to girls in the included (1:1) and excluded (1:2) study groups were different, with 37 girls being excluded from the study. The mean BMI z-scores for excluded and included children were − 0.28(1.81) and 0.07(0.9) respectively. (Table 1S1, Supplementary file 1).

### GLI_2012_ z-scores

The Shapiro Wilk test, highlighted that the FEV_1_/FVC (for both sexes) and MMEF (for boys) z-scores generated from our sample were not perfectly normally distributed (mean≠0, SD ≠ 1; Table 2) [39]. Nonetheless, the GLI2012 SRE for a given age, sex, height and ethnicity showed Q-Q plots in a straight line (Figure 1S2, Supplementary file 2) which indicated relative normality, although mean GLI SRE z-scores were negative. Importantly, the distribution of spirometry z-scores showed that the African-American module defined in the GLI_2012_ SRE is a good fit for urban and peri-urban Zimbabwean children. The African-American module gave the smallest absolute differences (closest to zero) as compared to other GLI_2012_ ethnic modules which were also generally out of the range of ±0.5.

**Table 2:** Mean GLI_2012_-z-scores for FVC, FEV_1_, FEV_1_/FVC ratio, MMEF by different ethnic GLI_2012_ modules

Spirometry	African American	Caucasian	North-Eastern Asia	South-Eastern Asia	Other-Ethnic Group	*p*-value
FVC						
*Boys*	−0.08 (0.97)	−1.41 (0.91)	−1.75 (1.49)	−0.49 (0.98)	− 0.82 (1.03)	0.075
*Girls*	−0.15 (1.05)	−1.38 (1.01)	−1.43 (1.24)	−0.21 (1.10)	− 0.80 (1.14)	0.217
FEV_1_						
*Boys*	−0.33 (0.90)	−1.57 (0.84)	−1.97 (1.30)	−0.90 (0.90)	− 1.05 (0.90)	0.134
*Girls*	−0.40 (0.99)	−1.56 (0.93)	−1.47 (0.96)	−0.63 (1.00)	− 1.05 (1.00)	0.480
FEV_1_/FVC						
*Boys*	−0.42 (1.11)	−0.29 (1.10)	− 0.46 (1.35)	−0.87 (1.24)	− 0.48 (1.16)	< 0.001
*Girls*	−0.43 (1.21)	− 0.34 (1.20)	− 0.55 (1.36)	−0.77 (1.16)	− 0.54 (1.25)	< 0.001
MMEF						
*Boys*	−0.48 (1.06)	−1.10 (1.17)	−1.12 (1.37)	− 1.18 (1.31)	− 0.89 (1.20)	< 0.001
*Girls*	−0.66 (1.02)	− 1.27 (1.11)	− 1.00 (1.20)	− 1.19 (1.20)	−1.08 (1.15)	0.458

*p*- value Test for normality using the Shapiro-Wilk test on the distribution of z-scores for the respective spirometry z scores by sex

*FVC* Forced vital capacity, *FEV*_*1*_ Forced expiratory volume in one second, *FEV1/FVC* Ratio of FEV_1_ to FVC, *MMEF* Maximal mid-expiratory flow

NOTE: All data in this table are presented as mean (Standard Deviation)

### Scatterplots and distribution of African-American GLI_2012_ z-scores

**Fig. 2:**
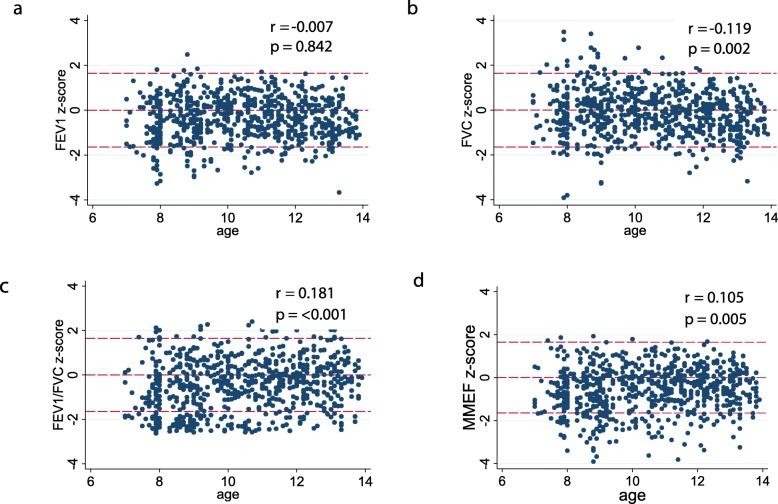
Scatterplots for GLI_2012_ z-scores for **a** FVC, **b** FEV_1_, **c** FEV_1_/FVC, **d** MMEF. Plots also demonstrate the distribution of the z-score values around 0, 1.645 and − 1.645

Scatterplots for spirometry z-scores did not show any linear trend (Fig. 2). The spread of z-scores was less variable for the FEV_1_/FVC ratio compared to FVC and FEV_1_ z-scores across age.

**Fig. 3:**
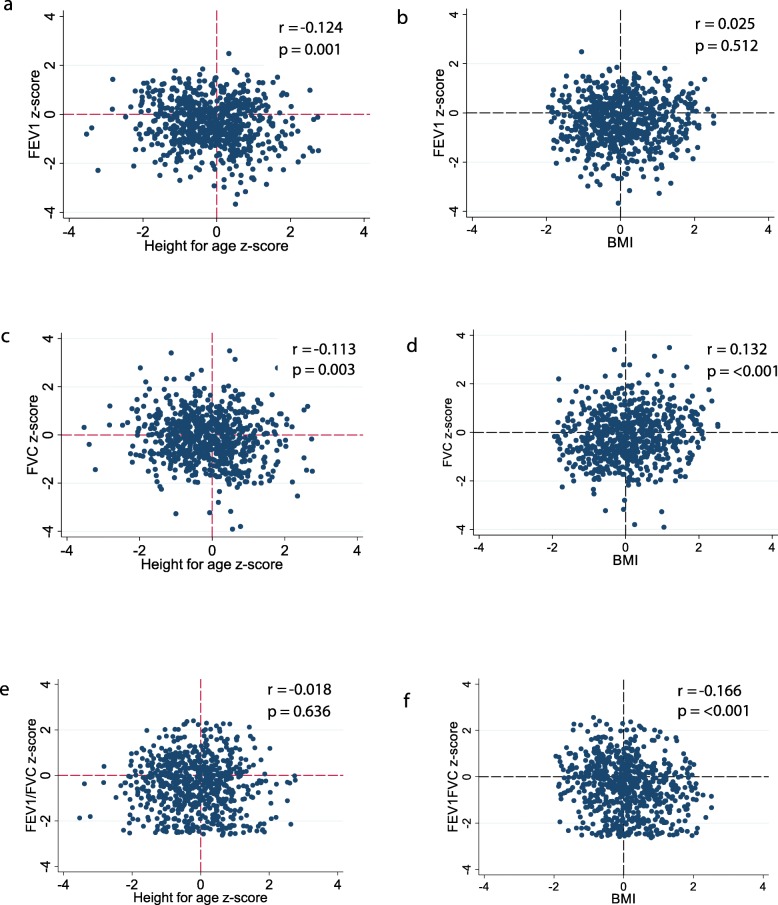
Scatterplots for **a**, **b** FVC, **c**, **d** FEV_1_, **e**, **f** FEV_1_/FVC z-scores against height and BMI z-scores

The scatterplots showed z-scores below the lower threshold values of − 1.64 (LLN) were not distributed in any particular pattern that might suggest an association of impaired lung function with age, height or BMI (Figs. 2 and 3). The distribution of spirometry z-scores in relation to the 5th percentile (LLN) identified that for FEV_1_, 8.7% (7.9% of boys, 9.6% of girls) and for FVC, 5.8% (4.1% of boys, 7.6% of girls) had values below the LLN. However, the FEV_1_/FVC z-scores showed a different pattern with 18.4% (18.2% of boys, 18.6% of girls) of children having values below the LLN indicating a deviation from the GLI_2012_ distribution.

### Anthropometric and demographic factors related to African-American GLI z-scores

The analysis of relationships between height, weight, BMI, age, and sex with spirometry z-scores demonstrated weak correlations, with Pearson’s correlation coefficient values between ±0.2 (Table 3). The linear associations between spirometry variables, anthropometric indices and school income as indicated by β coefficients from linear regression were within ±0.5 (Table 1S3, Supplementary file 3).

**Table 3:** Pearson’s correlation coefficients between spirometric variables and measured covariates

Spirometry variables (z-score)	Covariate			
	HAZ	WAZ	BMI Z	Age (years)
FVC	− 0.113(− 0.185–0.040)	0.022(− 0.052 0.095)	0.132(0.059 0.204)	−0.119(− 0.190–0.045)
FEV_1_	− 0.124(− 0.195–0.051)	−0.056(− 0.129 0.017)	0.025(− 0.049 0.098)	−0.007(− 0.066 0.081)
FEV _1_/FVC	− 0.018(− 0.091 0.056)	−0.122(− 0.193–0.049)	−0.166(− 0.237–0.094)	0.181(0.109 0.251)
MMEF	−0.053(− 0.126 0.020)	−0.068(− 0.141 0.006)	−0.057(− 0.130 0.016)	0.105(0.031 0.176)

*HAZ* Height for Age z-score, *WAZ* Weight for Age z-score, *BMI Z* Body Mass Index for age z-score calculated using the 1990 British anthropometric reference values

*FVC* Forced vital capacity, *FEV*_*1*_ Forced expiratory volume in one second, *FEV1/FVC* Ratio of FEV_1_ to FVC, *MMEF* Maximal mid-expiratory flow

NOTE: All values are correlations (95% confidence interval limits)

Scatterplots for spirometry z-scores plotted against BMI z-scores showed a central cluster around the origin (Fig. 3b, d, f), providing no evidence for bias. However, all height scatterplots (Fig. 3 a, c, e) were more dispersed across values of height z-score, suggesting greater variability compared to the BMI plots with this most evident for FEV_1_ across height z-scores (Fig. 3a). Scatterplots stratified by school showed similar patterns to unstratified plots showing no bias by SES. (Figure 1S4-3S4, Supplementary file 4).

### Comparison of the African-American GLI_2012_ and the Polgar SRE

**Fig. 4:**
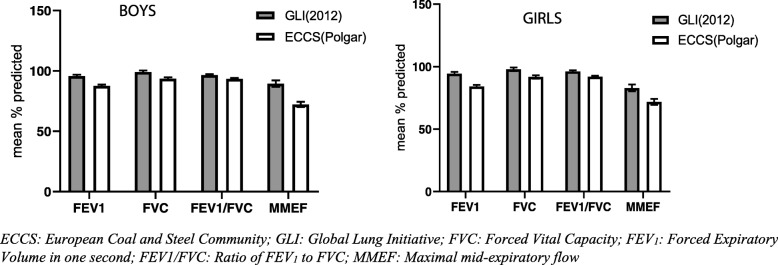
Mean values expressed as a percent of predicted values according to African-American GLI_2012_ and Polgar spirometric reference equations for **a** FEV_1_ **b** FVC **c** FEV_1_/FVC **d** MMEF

Comparisons between the mean percentage predicted for FVC, FEV_1_, FEV_1_/FVC and MMEF by sex, generated from the African-American GLI_2012_ and the Polgar SRE were performed. All of the mean percent predicted values were lower than 100% (full prediction) regardless of SRE used. Percent predicted values were consistently closer to 100% when using the GLI_2012_ as compared to the Polgar SRE, indicating a better fit for the African-American GLI_2012_ SRE. The FVC measurements were the least underestimated by the Polgar SRE whilst MMEF had the highest differences (Fig. 4). The observed patterns were the same in girls and boys. A Bland-Altman plot for the spirometric variables showed mean differences between the GLI_2012_ and Polgar SRE and evidence of proportional bias as the difference of GLI_2012_ and Polgar predicted values increased with the mean values of the two SRE. (Figure 1S5, Supplementary file 5: regression coefficients).

## Discussion

This study is the first to evaluate the use of the African-American GLI_2012_ SRE in Zimbabwean children aged 7–13 years attending primary school. Our findings demonstrate that lung function parameters for Zimbabwean children are comparable to those of African-American children as indicated by the overall fit of African-American GLI_2012_ SRE. Thus, the African-American GLI_2012_ SRE is applicable for use in Zimbabwean children.

These findings are consistent with other findings in children [15] and adults [40] from sub-Saharan Africa. The similarities in spirometric variables between Zimbabwean and African-American children highlight the influence of ethnic background on lung development in healthy individuals, regardless of healthcare access, exposure to air pollution and SES [15, 41, 42]. Indeed, we detected no difference in lung function patterns between schools belonging to areas characterised by a different SES in this study. We identified anthropometry differences in this population consistent with studies that have also highlighted sex-related differences in anthropometry and lung function indices in children of the same age [36, 37].

Z-scores for spirometry variables are dimensionless values that show the number of SDs the measurement is positioned from the GLI_2012_ SRE population values [2, 15]. The GLI_2012_ SRE predict standardised z-score values that are adjusted for ethnicity and anthropometric variables. Mean African-American GLI_2012_ z-scores for all the spirometry variables were within 0.5 z-scores from zero, which is within the acceptable range of the GLI_2012_ perfect fit prediction [15, 32]. However, the z-score SD for the FEV_1_/FVC ratio was ≥1, indicating more variability than the reference population, thus affecting the performance of the African-American GLI_2012_ LLN in this population [15, 43, 44]. By definition, the LLN allows 5% of healthy people to be misclassified and higher variability in FEV_1_/FVC may increase misclassification of airway obstruction [2, 44]. Conversely, however, as the overall population is slightly shifted down away from the predicted mean, this may reflect an actual reduction of FEV_1_/FVC in our population. The FEV_1_/FVC is sensitive to early life exposures and maybe an early indicator of decline in lung function later in life [45].

In this study, all the spirometry z-scores had a negative offset, indicating that the African-American GLI_2012_ SRE generates values which are slightly above those of Zimbabwean children regardless of sex. Mean predicted values for all spirometry values were lower than 100% (perfect fit), and the observed differences were lower in girls than boys.

With a perfect fit, the z-scores developed from the GLI_2012_ SRE should show a lack of association with ethnicity and anthropometric variables since they are independent variables for generating the LLN [8, 16]. We identified weak correlations between anthropometric and spirometry z-scores with no consistent direction. Furthermore, the scatterplots for these associations showed no particular pattern indicating a lack of any physiological correlations. Similar results indicating weak correlations were also reported in other studies from Tunisian, Swedish and Asian populations [10, 15, 16]. Analysis of the scatterplots and multivariable analysis stratified by school-income level showed inconsistent influence of SES in explaining the variability in lung function z-scores. However, the associations detected between FEV_1_/FVC and BMI z-scores may be contributing to the high variability in this measure, resulting in less goodness of fit by the African-American GLI_2012_ SRE. Furthermore, this finding highlights the possibility of more variability in the body frames of Zimbabwean as compared to African American children, and this may influence the association of anthropometric and spirometric measurements in our population.

Most physicians in Zimbabwe use the Polgar SRE for diagnosis of lung disease, which were developed from North America, Europe and Japan and compiled by Polgar & Promadaht (1971) for the 6–18-year age group [2, 34]. In contrast, the GLI_2012_ produced SRE from 74,117 healthy individuals worldwide. Mean comparisons of percent predicted GLI_2012_ SRE-derived values against the Polgar values in this population showed substantially higher lung function prediction for the African-American GLI_2012_ SRE (5.6, 9.1 and 3.6% in FVC, FEV_1_ and FEV_1_/FVC, respectively) [8, 46]. Results showing lower Polgar predicted values as compared to the GLI_2012_ values have also been identified in other populations [15, 46].

Our results suggest that the use of the African-American GLI_2012_ SRE in Zimbabwean children can improve identification of a tendency towards a restrictive and obstructive lung function pattern. Diagnosis of associated lung diseases can be enhanced by using LLN to identify impaired lung function rather than fixed-cut offs, as this approach mitigates the anthropometric and ethnic group related biases that can result in misclassification of borderline lung function [8, 47]. The LLN values were developed from a large sample using z-scores adjusted for ethnic groups, height, age and sex. The LLN values can help define lung function abnormality: airflow obstruction is defined as FEV_1_/FVC < LLN, whilst FEV_1_/FVC > LLN in combination with FVC < LLN can represent a tendency towards a restrictive pattern. Thus, it is possible that changing SRE from Polgar to African-American GLI_2012_ can alter the interpretation of spirometry results which will, in turn, affect the overall classification of patients as having a tendency towards an obstructive or restricted lung pattern, thereby, modifying the prevalence and subtypes of lung disorders [46, 48]. The negative mean spirometry z-scores for all the variables implies the LLN should be cautiously interpreted by practitioners, to avoid over-classifying children with low lung function.

This study represents a response to the call of the ERS to validate the GLI_2012_ SRE in ethnic groups that are not included in the sample used to derive these SRE [8]. Strengths of our study include a randomly selected sample, and high quality lung function variables collected in a standardised manner based on ATS/ERS guidelines. We used the same spirometer that was regularly calibrated to minimise variability, and the failure rate for valid measurements was low. We acknowledge several limitations. We had a 20% refusal rate but the overall sample size was sufficient to validate the GLI_2012_ SRE. The z-score calculations may have been subject to measurement error because they are adjusted for height which was measured only to the nearest centimetre; for instance, a one cm difference in height for a 12-year-old male child can relate to a difference of 0.08 and 0.1 in the predicted FEV_1_ and FVC z-scores, respectively. Our results may not be generalisable to other Zimbabwean settings where exposure to indoor and outdoor air pollution may differ from Harare; we did not measure air pollution so were unable to assess its effects. The study did not capture birthweight and preterm status which is associated with the general lung development in children.

## Conclusion

The African-American GLI_2012_ SRE are appropriate for predicting lung function in Zimbabwean school-going urban and peri-urban children aged 7–13 years. The use of the African-American GLI_2012_ SRE in healthy Zimbabwean children shows better prediction compared to the Polgar SRE, supporting that African-American GLI_2012_ SRE are the equations of choice to use in evaluating lung function in Zimbabwean urban and peri-urban school-age children.

## Supplementary information

**Additional file 1.** Comparison between children included and excluded from the study. A table summarizing the demographic and anthropometry characteristics for children included and excluded from the study.

**Additional file 2.** Visual plots. Histograms and Q-Q plots showing the distribution of anthropometric variables and spirometry z-scores.

**Additional file 3.** Regression analysis between spirometry and independent variables. Regression analysis showing the multivariable linear relationship between spirometry z –scores and independent variables (age, height, weight, BMI and SES).

**Additional file 4.** Scatterplots for anthropometric and spirometric z-scores. Scatterplots showing correlations between anthropometric and spirometric z-scores stratified by level of school income.

**Additional file 5.** Bland-Altman plots comparing the GLI_2012_ and Polgar SRE. The Bland-Altman plots for spirometry indices on FVC, FEV_1_, FEV_1_/FVC and MMEF comparing the performance of the GLI_2012_ and Polgar SRE in this sample.

## Data Availability

The datasets generated during and/or analysed during the current study are available from the corresponding author on reasonable request.

## Abbreviations

ATS: American Thoracic Society
COPDs: Chronic Obstructive Pulmonary Diseases
ECSC: European Coal and Steel Community
ERS: European Respiratory Society
FEV _1_: Forced Expiratory Volume in one second
FVC: Forced Vital Capacity
GLI_2012_: Global Lung Initiative 2012 equations
GOLD: Global Initiative for Chronic Obstructive Lung Disease
LLN: Lower Limit to Normal
MMEF: Maximal mid-expiratory flow of Forced Vital Capacity
PEF: Peak Expiratory Flow
PFTs: Pulmonary Function Tests
Q-Q: Quantile- Quantile
SES: socioeconomic status
SRE: Spirometric Reference Equations
WHO: World Health Organisation
