# Supplementary material for: Validation of the global lung initiative 2012 multi-ethnic spirometric reference equations in healthy urban Zimbabwean 7–13 year-old school children: a cross-sectional observational study
Source: BMC Pulm Med. 2020 Feb 28;20:56. doi: 10.1186/s12890-020-1091-4 (PMC7048020; doi:10.1186/s12890-020-1091-4)
Supplement: Supplementary file 1 — Additional file 1. Comparison between children included and excluded from the study. A table summarizing the demographic and anthropometry characteristics for children included and excluded from the study. [file 12890_2020_1091_MOESM1_ESM.docx]

**Table 1S1: Comparison between children included and excluded from the study**

| **Variables** | **Participants included (n=712)** | **Participants excluded (n=57)** | **p-value** |
| --- | --- | --- | --- |
| Age (years) | 10.5 (1.21) | 11.6 (1.45) | <0.001 |
| Sex n (%)  Boys  Girls | 368 (51.7)  344 (48.3) | 20 (35.1)  37 (64.9) | 0.016 |
| Height (cm) | 139.9 (10.36) | 151.04 (10.45) | <0.001 |
| Weight (kg) | 34.4 (7.73) | 42.4 (13.23) | <0.001 |
| BMI (kg/m^2^) | 17.4 (2.09) | 18.14 (4.20) | 0.010 |
| Height for age z-score | -0.15 (0.98) | 0.54 (1.22) | <0.001 |
| Weight for age z-score | -0.02 (0.89) | 0.15 (1.54) | 0.098 |
| BMI z-score | 0.07 (0.90) | -0.28 (1.81) | 0.011 |

*sd: standard deviation; cm: centimetres; kg: kilograms; BMI: body mass index*

*NOTE: All data in this table are presented as mean (SD)*
