# Supplementary material for: Validation of the global lung initiative 2012 multi-ethnic spirometric reference equations in healthy urban Zimbabwean 7–13 year-old school children: a cross-sectional observational study
Source: BMC Pulm Med. 2020 Feb 28;20:56. doi: 10.1186/s12890-020-1091-4 (PMC7048020; doi:10.1186/s12890-020-1091-4)
Supplement: Supplementary file 2 — Additional file 2. Visual plots. Histograms and Q-Q plots showing the distribution of anthropometric variables and spirometry z-scores. [file 12890_2020_1091_MOESM2_ESM.docx]

**Figure 1S2: Visual plots: histograms for anthropometry and Q-Q plots for spirometry z-scores**

*FEV_1_= Forced Expiratory Flow at one second; FVC= Forced Vital Capacity; FEV_1_/FVC = Ratio of FEV_1_ to FVC; MMEF=Maximal mid-Maximal Expiratory Flow; BMI: Body Mass Index: Q-Q; Quantile-Quantile*
