# Supplementary material for: Validation of the global lung initiative 2012 multi-ethnic spirometric reference equations in healthy urban Zimbabwean 7–13 year-old school children: a cross-sectional observational study
Source: BMC Pulm Med. 2020 Feb 28;20:56. doi: 10.1186/s12890-020-1091-4 (PMC7048020; doi:10.1186/s12890-020-1091-4)
Supplement: Supplementary file 3 — Additional file 3. Regression analysis between spirometry and independent variables. Regression analysis showing the multivariable linear relationship between spirometry z –scores and independent variables (age, height, weight, BMI and SES). [file 12890_2020_1091_MOESM3_ESM.docx]

**Table 1S3: Linear regression analysis for spirometry variables and categorical Socio-Economic Status (SES)**

| **Spirometry z-score** | **Weight(kg)** | **Height(cm)** | **Age(years)** | **SES** |  |
| --- | --- | --- | --- | --- | --- |
| FEV_1_ z-score | 0.02 | -0.03 | 0.06 | High Income  Medium Income  Low Income | 1(Reference)  -0.49  -0.12 |
| FVC z-score | 0.04 | -0.04 | 0.004 | High Income  Medium Income  Low Income | 1(Reference)  -0.39  -0.09 |
| FEV_1_/FVC | -0.04 | 0.03 | 0.09 | High Income  Medium Income  Low Income | 1(Reference)  -0.26  -0.09 |
| MMEF | -0.008 | -0.0007 | 0.074 | High Income  Medium Income  Low Income | 1(Reference)  -0.11  -0.31 |

*FEV_1_= Forced Expiratory Flow at one second; FVC= Forced Vital Capacity; FEV_1_/FVC = Ratio of FEV_1_ to FVC; MMEF=Maximal mid-Maximal Expiratory Flow; SES=Socio-Economic Status; p-value- Wald test for the homogeneity of the coefficients of SES in the multivariate analysis; SD= Standard Deviation; LLN=Lower Limit to Normal.*

*kg=kilograms; cm= centimetres;*

*NOTE: All values in the table are Beta coefficients.*
