# Supplementary material for: Validation of the global lung initiative 2012 multi-ethnic spirometric reference equations in healthy urban Zimbabwean 7–13 year-old school children: a cross-sectional observational study
Source: BMC Pulm Med. 2020 Feb 28;20:56. doi: 10.1186/s12890-020-1091-4 (PMC7048020; doi:10.1186/s12890-020-1091-4)
Supplement: Supplementary file 4 — Additional file 4. Scatterplots for anthropometric and spirometric z-scores. Scatterplots showing correlations between anthropometric and spirometric z-scores stratified by level of school income. [file 12890_2020_1091_MOESM4_ESM.docx]

**Figure 1S4: Scatterplots showing correlation between age and spirometry z- scores**

**Low Income** (n=267)

**Medium Income** (n=285)

**High Income** (n=160)

**Figure 2S4: Scatterplots showing correlation between height and spirometry z- scores**

**Medium Income** (n=285)

**High Income** (n=160)

**Low Income** (n=267)

**Figure 3S4: Scatterplots showing correlation between BMI and spirometry z- scores**

**Medium Income** (n=285)

**High Income** (n=160)

**Low Income** (n= 267)
