# Supplementary material for: Validation of the global lung initiative 2012 multi-ethnic spirometric reference equations in healthy urban Zimbabwean 7–13 year-old school children: a cross-sectional observational study
Source: BMC Pulm Med. 2020 Feb 28;20:56. doi: 10.1186/s12890-020-1091-4 (PMC7048020; doi:10.1186/s12890-020-1091-4)
Supplement: Supplementary file 5 — Additional file 5. Bland-Altman plots comparing the GLI2012 and Polgar SRE. The Bland-Altman plots for spirometry indices on FVC, FEV1, FEV1/FVC and MMEF comparing the performance of the GLI2012 and Polgar SRE in this sample. [file 12890_2020_1091_MOESM5_ESM.docx]

*FEV_1_= Forced Expiratory Flow at one second; FVC= Forced Vital Capacity; FEV_1_/FVC = Ratio of FEV_1_ to FVC; MMEF=Maximal mid-Maximal Expiratory Flow; CI: confidence interval*

**Figure 1S5: Bland Altman Plots comparing the performance of the GLI_2012_ and the Polgar spirometric reference equations**

Limits of Agreement = [1.272 27.380]

Mean difference = 14.326(CI: 13.845 14.807)

Regression coefficient = 0.17 (p-value: <0.001)

Limits of Agreement = [-0.398 11.779]

Mean difference = 5.690(CI: 5.466 5.914)

Regression coefficient= 0.04 (p-value: <0.001)

Limits of Agreement = [2.496 15.929]

Mean difference = 9.212(CI: 8.965 9.459)

Regression coefficient = 0.05 (p-value: <0.001)

Limits of Agreement = [-0.454 7.753]

Mean difference = 3.649(CI: 3.498 3.80)

Regression coefficient = 0.04 (p-value: <0.001)
